# NIR-to-NIR Imaging: Extended Excitation Up to 2.2 μm Using Harmonic Nanoparticles with a Tunable hIGh EneRgy (TIGER) Widefield Microscope

**DOI:** 10.3390/nano11123193

**Published:** 2021-11-25

**Authors:** Laura Vittadello, Jan Klenen, Karsten Koempe, Laura Kocsor, Zsuzsanna Szaller, Mirco Imlau

**Affiliations:** 1 Department of Physics, Osnabrueck University, 49076 Osnabrueck, Germany; laura.vittadello@uni-osnabrueck.de (L.V.); jklenen@uni-osnabrueck.de (J.K.); 2 Research Center for Cellular Nanoanalytics, Osnabrueck (CellNanOs), Osnabrueck University, 49076 Osnabrueck, Germany; karsten.koempe@uni-osnabrueck.de; 3 Department of Biology/Chemistry, Osnabrueck University, 49076 Osnabrueck, Germany; 4 Wigner Research Centre for Physics, Institute for Solid State Physics and Optics, Konkoly-Thege M. út 29-33, H-1121 Budapest, Hungary; kocsor.laura@wigner.hu (L.K.); szaller.zsuzsanna@wigner.hu (Z.S.)

**Keywords:** biological windows, NIR-III, NIR-IV, NIR imaging, nonlinear microscopy, nonlinear photonics, deep tissue imaging harmonic nanoparticles, second harmonic generation

## Abstract

Near-infrared (NIR) marker-based imaging is of growing importance for deep tissue imaging and is based on a considerable reduction of optical losses at large wavelengths. We aim to extend the range of NIR excitation wavelengths particularly to values beyond 1.6 μm in order to profit from the low loss biological windows NIR-III and NIR-IV. We address this task by studying NIR-excitation to NIR-emission conversion and imaging in the range of 1200 up to 2400 nm at the example of harmonic Mg-doped lithium niobate nanoparticles (i) using a nonlinear diffuse femtosecond-pulse reflectometer and (ii) a Tunable hIGh EneRgy (TIGER) widefield microscope. We successfully demonstrate the existence of appropriate excitation/emission configurations in this spectral region taking harmonic generation into account. Moreover, NIR-imaging using the most striking configurations NIR-III to NIR-I, based on second harmonic generation (SHG), and NIR-IV to NIR-I, based on third harmonic generation (THG), is demonstrated with excitation wavelengths from 1.6–1.8 μm and from 2.1–2.2 μm, respectively. The advantages of the approach and the potential to additionally extend the emission range up to 2400 nm, making use of sum frequency generation (SFG) and difference frequency generation (DFG), are discussed.

## 1. Introduction

**Figure 1:**
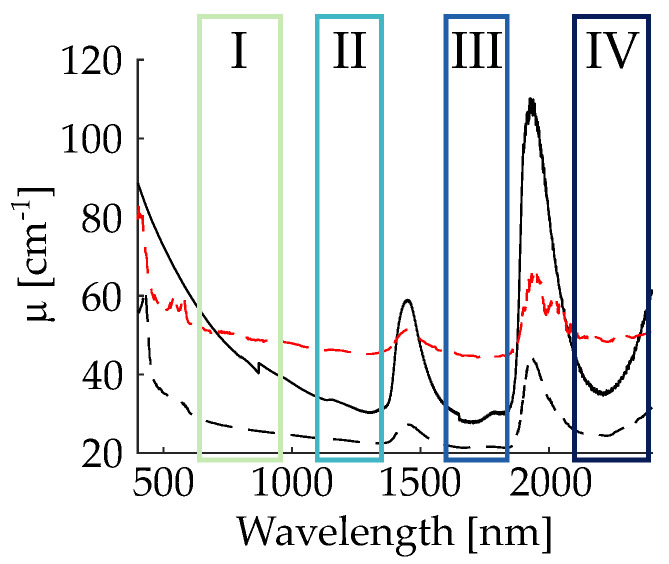
Extinction coefficient of PMMA nano-spheres diluted in water (black solid line) used as phantom tissue, skin (black dotted line) and skull bone (red dotted line). Superposed to the spectra, the information on the biological windows NIR-I, II, III, IV are added. (Spectra of skin and skull bone are taken from Ref. [11]).

Near-infrared, marker-based imaging (NIR, 700–2500 nm) is of growing interest for deep tissue imaging [1]. It benefits from considerably reduced light scattering, residual absorption and disappearance of autofluorescence phenomena in tissue and, thus, potentially permits us to obtain high-contrast images at the lowest possible light-induced tissue damage [2,3]. Nowadays, a variety of NIR-to-NIR biomarkers for the use in state-of-the-art microscopic setups exist (using NIR light for the excitation and emission) [4,5,6,7], that can be excited at wavelengths in the biological windows NIR-I (650–950 nm) and NIR-II (1100–1350 nm) where the tissue extinction coefficient is lower compared with the visible (VIS) (see Figure 1). In contrast, so far, there are only a few studies that make use of the NIR-III (1600–1850 nm) and NIR-IV (2100–2300 nm) biological windows. Studies in brain tissue [8], skin [9] and bones [10] show that there is a much lower light extinction coefficient in these two wavelength regions compared with NIR-I and the VIS.

Shifting the interest towards the NIR-III and NIR-IV windows is challenging because not only biomarkers that can be excited here are needed, but also on the detection side, a suitable camera system is needed if the emission appears in this spectral range [12]. In this article, we address this task by studying nonlinear NIR-to-NIR imaging at the example of harmonic nanoparticles (HNPs) in a Tunable hIGh EneRgy (TIGER) widefield microscope [13] with a particular focus on the excitation within the NIR-III and NIR-IV biological windows, i.e., a marker-based approach in multiphoton microscopy. While marker-free (unlabeled) microscopy has emerged already as powerful tool in medical–technical applications, the development of nanoparticle-based approaches aims at molecular monitoring, optogenetics and drug release [14,15], as it has been recently successfully demonstrated with harmonic LiNbO${}_{3}$ [16]. In this context, harmonic nanoparticles profit not only from the controlled generation of high-energy photons at the molecular scale, but also from the high spectral selectivity of emission, as demonstrated in [17]. In general, the use of marker-based multiphoton microscopy is not very well explored thus far. Indeed, only recently, we demonstrated the possibility for tracking HNPs in-vivo in the heart of a living *Drosophila* larvae with this concept using 1200 nm for excitation (NIR-II) and 600 nm (VIS) for detection. For the present study, we make use of the large tuning range of the laser system from 630–2700 nm without gap and the continuous nonlinear optical response of the HNPs, i.e., nonlinear optical theory imposes no constrains to the nanoparticles excitation and/or emission in these spectral ranges [18].

We show that the combination of harmonic nanoparticles and tunable high-energy laser system of the TIGER microscope possess all the required condition for NIR-imaging beyond the NIR-II window: it can either be optically excited till 2400 nm, i.e., covering the biological windows NIR-II, NIR-III and NIR-IV, and generates emission from 400–1200 nm, i.e., covering the NIR-I and NIR-II biological windows. We further show NIR-to-NIR-imaging using the two striking configurations NIR-III to NIR-I and NIR-IV to NIR-I, the latter for the first time, to best of our knowledge at excitation wavelengths of up to 2.2 μm. We discuss our findings in the framework of commercially available microscopes and laser systems. Moreover, the generation of NIR emission by means of sum frequency and difference frequency generation is addressed. As a result, wavelengths of up to 2400 nm can be generated in principle by mixing two pulses of different colors, thus, enabling emission in the NIR-III and NIR-IV biological windows.

The paper is organized as follows: in the first step, the concept of nonlinear optical excitation and emission in the NIR-II, NIR-III and NIR-IV is systematically inspected by means of powder pellets of harmonic Mg-doped lithium niobate nanoparticles and nonlinear diffuse femtosecond-pulse reflectometry. Tuning the pump wavelength from 1200–2400 nm without any gap demonstrates the principle validity of the approach and is used to select the most promising wavelength pairs of fundamental and second/third harmonic light for NIR-to-NIR imaging. In the second step, the respective wavelength parameters are transferred to the TIGER microscope. Here, the concept is validated with dried powder samples on a cover slip, but also for the case that phantom tissue is present in the optical path. The study is used to validate NIR-III to NIR-I, as well as NIR-IV to NIR-I imaging with excitation wavelengths in the range 1.6–1.8 μm and 2.1–2.2 μm, respectively. Moreover, a direct comparison of tissue imaging with state-of-the-art wavelength pairs is used in order to evaluate the added value of the concept. The results are discussed, and a perspective for NIR emission up to 2400 nm based on the mixing of two colors of different colors in the harmonic nanoparticles according to the nonlinear optical schemes of sum frequency generation and difference frequency generation is given.

## 2. Materials and Methods

### 2.1. Sample Prepearation

The harmonic nanoparticles are prepared from a magnesium-doped lithium niobate (LN:Mg) single crystal (6.5mol% MgO in the melt, grown from the congruently melting composition) via high-energy ball-milling [19]. A nanoparticle powder sample with a size distribution centered at ≈90 nm was obtained. Part of the sample was used for nonlinear optical characterization by means of nonlinear diffuse fs-pulse reflectometry. For this purpose, the nanoparticle powder was pressed to form a solid pellet, as described in detail in Ref. [20]. For our studies using the nonlinear optical TIGER microscope, the nanoparticles were mixed with water, dropped onto a standard cover slip, and dried at room temperature. The nanoparticles adhered on the substrate without any further chemical support. They were then sealed by another cover slip and mounted in a custom-design chamber, which allowed us to add a column of phantom tissue with a height of up to 0.6mm on the bottom. The phantom tissue was created by mixing PMMA (polymethyl methacrylate) (Sigma-Aldrich Chemie GmbH, Merck, Taufkirchen, Germany) spheres having a mean hydrodynamic diameter of 450 nm with water [21]. The final solution had a PMMA concentration of 13.2 mg/mL. This value is chosen in order to reproduce the optical fingerprint of the skin according to results reported in [11]. The result of the extinction coefficient is visible Figure 1.

### 2.2. Nonlinear Diffuse Femtosecond-Pulse Reflectometry

Nonlinear diffuse femtosecond-pulse reflectometry is applied to characterize the nonlinear optical properties of the as-synthesized HNPs [22]. Briefly, the pulse train of a regeneratively amplified femtosecond (fs) pulse laser system, model: Pharos-HE-20 (Light Conversion Inc., Vilnius, Lithuania ), with optical parametric amplifier (OPA), model: Orpheus F (Light Conversion Inc., Vilnius, Lithuania), is tightly focused at a small angle of incidence onto the nanopowder pellet. Diffusively emitted light is detected in reflection geometry and at an angle out-of the specular reflected beam using a set of a visible (VIS) and near-infrared (NIR) fibre spectrometers. In more detail, the Idler (λ = 1030–2400 nm) configuration of the OPA is used at a repetition rate of 50kHz with sub 100-fs laser pulses for all measurements. A longpass filter (FEL1150, Thorlabs Inc., Newton, NJ, USA) blocks the residual pump (515nm) and signal (λ = 630–1030 nm) radiation at the exit of the OPA. A tunable ND-filter wheel (NDC-50C-2, Thorlabs Inc., Newton, NJ, USA) serves for adjustment of the pulses energy in the range from 0.2 to 3.9 μJ. The pulse train is focused onto the nanoparticle powder-pellet with a plano-convex lens (f = 150mm) placed at a distance of 160mm in front of the sample’s surface. In order to acquire the diffusely reflected radiation, two fibre spectrometers, model types: QEPro and NIRQuest (Ocean Optics Inc., Ostfildern, Germany), are used, covering the spectral ranges of the VIS (266–1030 nm) and NIR(1150–1720 nm), respectively. This makes the validation of pairing of fundamental excitation and second harmonic emission in all three combinations of biological optical windows possible: NIR-II/NIR-I, NIR-III/NIR-I, and NIR-IV/NIR-II. The fibers enable the geometric separation between diffusive and specular reflected light. The fiber aperture is positioned at the same height of the incident laser light and is directed to capture light propagating at the smallest possible angle with respect to the sample’s surface normal vector. The spectrometer’s integration times are chosen in the range from 100ms to 40s to optimize the signal yield. All spectra are corrected for the baseline. The VIS and NIR spectra were then merged to end-up with a single spectrum spanning from 266 up to 1720 nm, and subsequently, each spectrum’s most prominent peak was used for normalization. 2D color plots with a logarithmic scale for the intensities are used to visualize spectral features at highest possible contrast.

### 2.3. Tunable High-Energy (TIGER) Widefield Microscopy

Widefield nonlinear optical imaging is performed using the TIGER microscope introduced in Ref. [13]: a tunable, high-energy fs-pulse laser system (identical to the one used for nonlinear diffuse fs-pulse reflectometry) is collinearly coupled via a beam stabilization system, model type: Aligna 4D (TEM Messtechnik GmbH, Hannover, Germany), with the widefield illumination beam path of an inverted microscope (type: FV3000, Olympus Europa SE & Co. KG, Hamburg, Germany. Excitation is possible with wavelengths in the range from 630–2500 nm, thus covering all four biological optical windows NIR-I, NIR-II, NIR-III, and NIR-IV. A filter cage transmits and filters the light via exchangeable band pass filters to an Hamamatsu Photonics K.K. sCMOS camera sensor, model type: ORCA Flash 4.0 V3, that is optimized for detection in the VIS spectral range and, therefore, is limited to wavelengths at a maximum of 1000nm in the NIR, i.e., light in the biological optical window NIR-I can be detected. This makes pairing of fundamental excitation and second/third-harmonic emission possible either using NIR-II/NIR-I or NIR-III/NIR-I and, respectively, NIR-IV/NIR-I. For all studies, the repetition rate is set to 50kHz that is large enough to obtain images at sufficiently large signal to noise (SNR) ratio (SNR ≫10) and low enough to suppress laser damage of the biological tissue. For concept validation using nanoparticle powders on a cover slip (see Section 3.2) a 60× water immersed objective (UPLSAPO, Olympus Europa SE & Co. KG, Hamburg, Germany, numerical aperture NA=1.2) is used. This objective obeys a custom coating to ensure light transmission in the VIS and NIR up to a wavelength of 1400nm, i.e., second harmonic emission in NIR-I and NIR-II can pass without considerable losses. The camera integration time is varied between 5 and 9 s, and the pulse peak energy is chosen in the range of 1–5 μJ to optimize the signal yield. The illuminated area is tailored with a plano-convex lens (f=150mm) to match with the objective’s field of view. For the validation of the HNP emission in combination with the phantom tissue (see Section 3.2), a 10× objective (UPLANFI, Olympus Europa SE & Co. KG, Hamburg, Germany, NA=0.3) is used. Here, the laser intensity is adjusted to $1.4\pm 0.3\times 10^{12}\;W/m^{2}$ for both wavelengths used and the camera integration time is fixed to 2 s.

## 3. Results

### 3.1. Nonlinear Diffuse Femtosecond-Pulse Reflectometry

**Figure 2:**
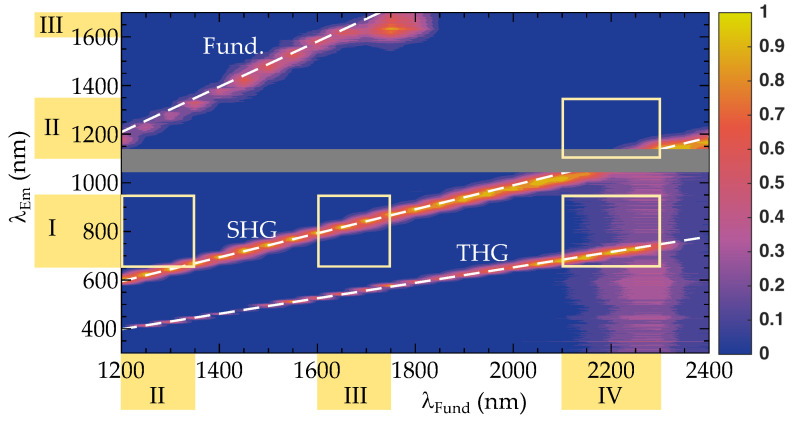
Normalized intensity (logarithmic scale) of harmonic emission in false colors in the wavelength range from 266–1720 nm as a function of the pump wavelength in the range from 1200–2400 nm. All data were obtained by means of nonlinear diffuse fs-pulse reflectometry with LN:Mg nanoparticle powder pellets, as described in the text. Color coding according to the legend on the right. The greyish area marks the spectrometers dead zone, i.e., the transition regime between the VIS and NIR spectrometer. The dashed white lines shows the result of the fitting procedure. The yellow boxes represent the principle regimes for NIR-to-NIR pairing of the biological optical windows NIR-II/NIR-I, NIR-III/NIR-I, NIR-IV/NIR-I, and NIR-IV/NIR-II.

Figure 2 shows the results of the nonlinear diffuse fs-pulse reflectometry measurements. The 2D plot highlights the intensity of the diffuse emission in false colors (color coding for the intensity at a logarithmic scale is shown to the right) in the spectral range from $300<\lambda_{\mathrm{SHG}}<1700$ nm upon excitation with wavelengths in the range of $1200<\lambda_{\mathrm{pump}}<2400$ nm. As an easy guide-to-the-eye, the red-orange-yellow colored areas refer to intensities much larger than the signal noise.

The 2D-plot was assembled from a series of diffusive emission spectra determined at fundamental pump wavelengths in steps of $\Delta \lambda_{\mathrm{fund}}=50$ nm. Residual scattered light of the incident fundamental pulse is detected in the range from 1200–1600 nm (weak linear feature in the upper left part of the figure). Intense diffuse emission appeared showing two distinct features from 600–1200 nm and from 400–800 nm. The small gap from 1030 to 1150 nm (greyish shaded area) is due to a dead detection zone arising from the missing spectral overlap of the VIS and NIR spectrometers (see Section 2.2 for more details). The characteristics of the emission unambiguously point to second (SHG) and third (THG) harmonic generation at their origin: the line width is smaller than 30 nm and, thus, much smaller than is common for luminescence. Most striking is the linear relationship between the fundamental excitation and harmonic emission wavelengths with slopes of 0.49±0.01 (SHG, theoretical value: 1/2) and 0.32±0.01 (THG, theoretical value: 1/3) that were determined from linear fits to the experimental data set. For comparison, the white dashed lines in Figure 2 represents the result of the fitting procedure. The dependence of the SHG intensity with respect to fundamental power in LiNbO${}_{3}$ has previously been investigated in ref. [23] and was found in accordance with our investigations using the TIGER microscope for KNbO${}_{3}$ [13], but also from other authors and harmonic nanoparticles (cf. BaTiO${}_{3}$ [24] and BFO [25]) with similar nonlinear optical properties to LiNbO${}_{3}$ [26]. In particular, a quadratic dependence of the SHG signal with respect to the power of the fundamental is reported. There are two more emission features that show weak emission over broad spectral ranges of different origin: one is found all over the visible spectral range for the case of large fundamental wavelengths between 2200 and 2400 nm. This feature can be attributed to the low pulse energy of the pump that results in a weak signal to noise ratio (SNR). The second one appears in the regime 1700–1850 nm. Here, the fundamental radiation is clipped by the spectrometer detection range. As a consequence, the normalization process results in a distorted representation of the short wavelength edge of the fundamental pulse spectrum. Both features will thus not be discussed any further.

The yellow squares in the figure highlight the spectral region where the wavelengths of the excitation–emission pair fall into biological optical windows. Obviously, SHG can be used with a considerably large tuning range for the pair NIR-III/NIR-I, and to some extent also for NIR-II/NIR-I and NIR-IV/NIR-II. Accordingly, THG is of interest for the pairing NIR-IV/NIR-I.

### 3.2. Tunable High-Energy (TIGER) Widefield Microscopy

**Figure 3:**
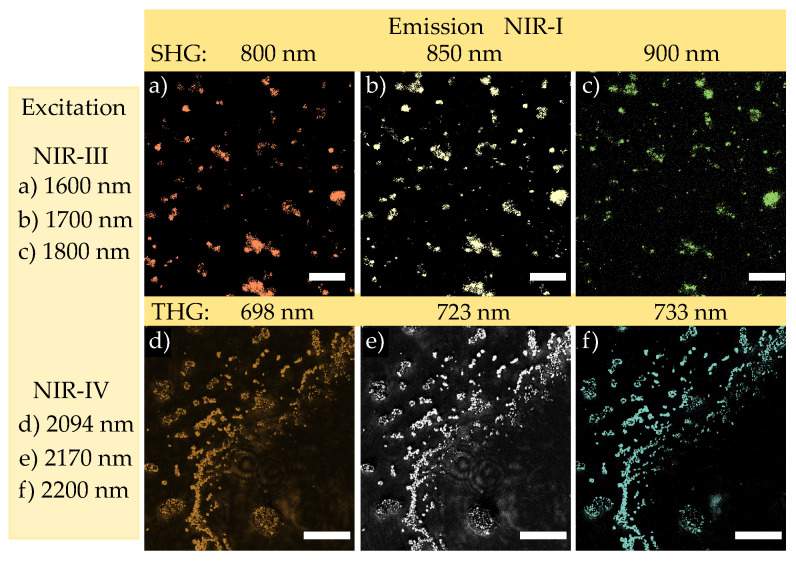
Nonlinear images acquired from nanoparticles dried on a coverslip using the TIGER microscope. NIR-III/NIR-I configuration using SHG: (**a**) 1600/800 nm (**b**) 1700/850 nm (**c**) 1800/900 nm. NIR-IV/NIR-I configuration using THG: (**d**) 2094/698 nm (**e**) 2170/723 nm and (**f**) 2200/733 nm. Scale bar is equal for all pictures to 20 μm. Images are presented in pseudo-color.

Figure 3 shows the results for imaging of harmonic nanoparticles dried on a cover slip using the TIGER microscope for excitation/emission configurations that fit with two pairs of biological windows: NIR-III/NIR-I (Figure 3a–c) and NIR-IV/NIR-I (Figure 3d,e). These configurations were chosen according to the findings of Figure 2 and the consideration of the spectral features of the CMOS sensor array of the camera. The latter is limited to the detection of emission in the biological window NIR-I.

In particular, Figure 3a–c show the images that were obtained under exposure to wavelengths of 1600, 1700 and 1800 nm, respectively. All images represent the same spatial distribution of the emitted light emerging from different regions within the field of view. However, different emission wavelengths were validated using different bandpass filters placed in the beam path of the FV3000 microscope. They coincide well with the expected SHG emission of 800, 850 and 900 nm. The images thus resemble the spatial distribution of nonlinear emission via SHG according to the NIR-III/NIR-I configuration. They are also identical to the spatial distribution of the as dried nanoparticles on the cover slip. In addition, our results show that the emitted signal intensity of the harmonic nanoparticles is sufficient for detection with commercial CMOS camera systems. This means that the frequency conversion efficiency from the ratio of excitation and emission intensity is sufficient for this application. However, a quantitative value based on our data cannot be determined. It is due to the fact, that the calculation of the overall efficiency depends strongly on the nanoparticle orientation, size and wavelengths. In our case, the samples are either nanopowder or small nanoparticle clusters, i.e., a composition of randomly oriented nanoparticles that contribute to the emitted signal must be assumed in both cases. In addition, different wavelengths sets were used, i.e., dispersion features of the conversion efficiency need to be considered, as well. However, studies at individual harmonic nanoparticles and for discrete wavelength sets exist, such as the calculation of the the two-photon cross-section value for ${\mathrm{BaTiO}}_{3}$ nanoparticles at 800 nm [27]. In [26], it was shown that the nonlinear averaged tensor elements measured at 1064 nm are in the same order of magnitude for ${\mathrm{BaTiO}}_{3}$ and ${\mathrm{LiNbO}}_{3}$. Taking into account the dispersion properties of the nonlinear optical susceptibility [28], it is to be expected that the cross section will be in the similar order of magnitude to the one calculated by [27]. At the same time, it should be noted, that nonlinear images obey a few characteristics. For instance, intensity differences of the SHG emission are due to different sizes of the nanoparticles and/or the presence of nanoparticle clusters as well as the nanoparticles orientations with respect to the light polarization. However, it should be noted that the latter effect is known to be particularly crucial in the case of single nanoparticle investigations [24,27,29,30], whereas in the present investigation the former two effects are of larger significance.

A similar situation is present in Figure 3d–f with the difference that the detected emission refers to THG emission at 698, 723 and 733 nm when exposed to 2094, 2170 and 2200 nm, i.e., the NIR-IV/NIR-I configuration.

**Figure 4:**
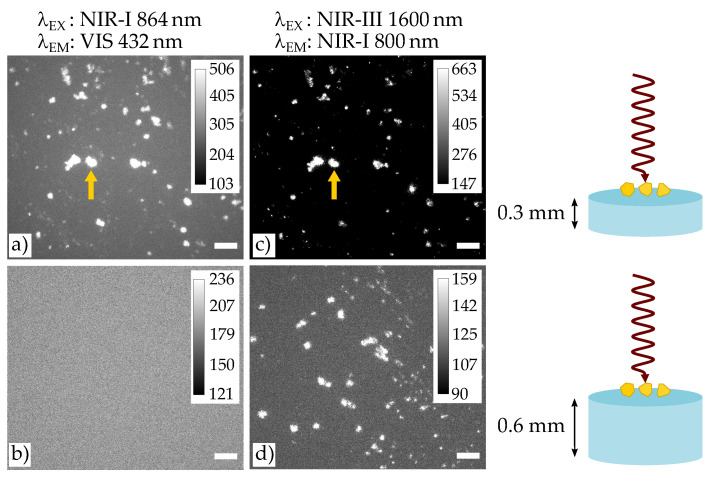
Study of the HNP emission in presence of a phantom tissue thickness of 0.3 mm (**a**,**c**) and 0.6 mm (**b**,**d**) for two different excitation wavelengths belonging, respectively, to NIR-I (**a**,**b**) and NIR-III (**c**,**d**). Scale bar is 50 μm for all figures.

In the next step, the role of deep tissue imaging is studied considering the previous findings (cf. Figure 4). Focus is given to the particular limit case, where nonlinear imaging using the common NIR-I/VIS configuration becomes weak or even fails, as shown in Figure 4a,b. Here, the excitation wavelength is adjusted to 864 nm, so that emission based on SHG is to be expected at 432 nm. Two different thicknesses of the phantom tissue were used: with a thickness of 0.3 mm in (a) and with a thickness of 0.6 mm in (b). Note, that the particles were placed on the top of the phantom tissue such that the SHG emission was forced to transmit through the entire sample thickness (see also sketches on the right in Figure 4). While image (a) allowed for the validation of the emission wavelength of 432 nm and shows the particle distribution weakly, but still visible, it is not visible at all in (b), i.e., for the case of the thick sample. Since only the thickness was changed as a parameter, this result must be attributed to the exponential damping of the SHG emission according to Beer’s law.

The same study was repeated using excitation/emission wavelengths of the NIR-III/NIR-I configuration. Figure 4b,d show the related images. In both cases, the wavelength of 800 nm was validated and the spatial distribution of the harmonic nanoparticles is resembled well. It is noteworthy that the situation in (c) already shows a striking advantage over the results of (a): the SNR of the cluster marked by the yellow arrow has been improved to a large extend from SNR = 94 in (a) up to an SNR = 234 in (c). Obviously, the major reason for this improvement is the lower extinction coefficient of the tissue at the emission wavelength of 800 nm, that allows for transmission with sufficient signal at the detector even for the thick sample. Note, that this situation becomes possible by pairing with NIR-III excitation and the use of harmonic generation, only.

## 4. Discussion

Our study includes some specific findings in the field of NIR imaging based on harmonic generation that can be used as a basis for a broader consideration of the topic. In the first part of the discussion, we will therefore analyze the results obtained from nonlinear diffuse fs-pulse reflectometry and NIR microscopy using the TIGER setup from the point of view of SHG and THG. Based on these findings, we will then map out a perspective for further studies and, above all, applications in the second part. In this context, we will consider further nonlinear optical schemes, such as sum frequency generation and difference frequency mixing. The aim is to contribute to the evaluation of the added value of the presented concept of NIR-to-NIR imaging in a wider context, even if it is limited to the application of polar oxide harmonic crystals in this work.

### 4.1. Nonlinear Diffuse fs-Pulse Reflectometry

Let us start with the results of the nonlinear diffuse fs-pulse reflectometry according to the results of Figure 2. Obviously, our data demonstrate the applicability of HNPs as nanoscopic light emitting markers far into the near-infrared spectral region. Indeed, the nanoparticles can be excited in a continuous way from 1200 nm to 2400 nm without any gap, thus generating emission spanning from 400 up to 1200 nm via second and third harmonic generation. This finding is very remarkable and, moreover, in full accordance with textbook knowledge in theoretical nonlinear optics [18]: the nonlinear response of polar harmonic nanoparticles is determined by the frequency-dependencies of the susceptibility of second and third order that are non-zero all over the optical and near-infrared spectral region. The respective dispersion is determined by the principle behavior of frequency dependence of the normal dispersion taking perturbation theory into account [31,32,33]. Thus, nonlinear optical emission may only break-down in the vicinity of pronounced (multi-)photon absorption, either due to the presence of dopants and/or impurities, or for photon energies close to resonance [34]. In polar oxides, the latter is determined by the energy gap of the band-to-band transition, that is 4.1 eV for the wide-band gap LN crystals [35]. The former can be excluded for the nanoparticles used in our study, as Mg-doping has no optical transitions in the investigated spectral range. At the contrary, Mg-doping supports to increase the optical quality of LN as Nb${}_{\mathrm{Li}}$ anti-site defect centers, usually giving rise to light-induced absorption [36,37,38], are efficiently suppressed, i.e., almost stoichiometric LN nanocrystals are obtained.

By far more interesting is the particular aspect of accessible excitation/emission configurations in all possible and application-relevant combinations of NIR-I, NIR-II, NIR-III and NIR-IV. It is noticeable that under the aspect of second and third harmonic generation a few combinations fail directly despite the large tuning range of harmonic nanoparticles and according to Figure 2. These are: NIR-II/NIR-II, NIR-III/NIR-II, NIR/III-NIR-III, NIR-IV/NIR-III and NIR-IV/NIR-IV. In contrast, the two most promising configurations are NIR-III/NIR-I (SHG) [39] and NIR-IV/NIR-I (THG). With strong limitations, also NIR-II/NIR-I (SHG) and NIR-IV/-NIR-II (SHG) can be used. The former is already well established [40,41,42], the latter requires an NIR sensitive sensor array, such as based on InGaAs semiconductors.

### 4.2. NIR-III to NIR-I and NIR-IV to NIR-I Imaging Based on the TIGER Microscope

The application of our findings to the TIGER microscope underlines the validity of our considerations for NIR-to-NIR imaging applications, and particularly underlines the impact of the two striking NIR-III/NIR-I (SHG) and NIR-IV/NIR-I (THG) configurations. As predicted from the results of Figure 2, nonlinear optical images are obtained with comparable high contrast at all inspected NIR wavelengths. It is noteworthy that the used laser energy density of $0.3\;J/m^{2}$ (NIR-III) and $0.2\;J/m^{2}$ (NIR-IV) were much below the damage threshold limits ($10^{4}\;J/m^{2}$ for NIR-III and $10^{3}\;J/m^{2}$ for NIR-IV [43]). This also means that it is still possible to increase the signal to noise ratio by several orders of magnitude, i.e., to increase the final image contrast, if at all necessary. To the best of our knowledge, these are the first images based on harmonic markers in this spectral range, and, moreover, the first images of markers that are pumped at wavelengths larger than 2 μm. As the emission of both configurations fit well with the spectral sensitivity of the sensor array of the camera, these configurations may facilitate the use of nonlinear optical imaging studies in biological tissue. Moreover, the possibility to couple the output of commercially available OPA systems to the widefield beam path of an inverted microscope in an easy manner will strongly support the installation of appropriate systems in laboratories.

At this point, it becomes necessary to discuss the advantages of the TIGER configurations in direct comparison with existing commercial nonlinear optical microscopes, commonly denoted as multi-photon microscopes. Such systems are in use over decades in bio-optical laboratories and make use of the tuning ability of mode-locked Ti:sapphire laser systems in the spectral region from 650 to 1100 nm. Meanwhile, this spectral region is extended up to 1350 nm (and even up to 3750 nm, see below), in full accordance with the rising needs to additonally cover the NIR-II biological window for optical excitation. As a matter of fact, the power map of commercial Ti:sapphire laser systems feature a maximum gain at about 800 nm, which is, therefore, the most commonly used wavelength region in biological studies. Accordingly, the fundamental wavelength in Figure 4a,c was chosen to 864 nm, to enable the common NIR-I/VIS (SHG) configuration with the CMOS sensor. In comparison, the wavelength 1600 nm is chosen for the fundamental wave in order to match the NIR-III/NIR-I configuration. As a result, the image quality is in favor of the NIR-III/NIR-I configuration. However, more striking is the fact, that NIR-I/VIS fails for imaging if the tissue under study exceeds a certain thickness. In other words, the commonly applied multiphoton microscopy technology prohibits optical access to samples at certain thicknesses. Or, as the product of thickness and extinction coefficient plays the major role in Beer’s law, optical access to materials with certain extinction coefficients is prohibited. In this sense, particular investigations of biological tissue are possible only, if the concept of NIR imaging in the biological windows NIR-III and/or NIR-IV is used.

At first sight, mode-locked Ti:sapphire laser systems with extended spectral ranges using OPOs seem to be a promising alternative in this respect. Already, integrated systems are commercially available that enable emission up to 3750 nm and show only a small gap at about 1700 nm. However, these systems unfortunately cannot be applied for applying the NIR-III/NIR-I configuration due to the inherent large average power in the order of 0.1–1 Watt at a high repetition rate (typically 80 MHz). It results in laser-damage of the tissue as well as in a weak conversion efficiency in the harmonic nanoparticle. We would like to emphasize that, for this reason, the repetition rate of the TIGER is reduced to 50 kHz, while delivering a sufficient pulse peak energy density (of at max 0.3 J/m${}^{2}$ in NIR-III), i.e., the average power is kept in the milli-Watt region.

### 4.3. Perspective of Our Findings for NIR-Microscopy

**Figure 5:**
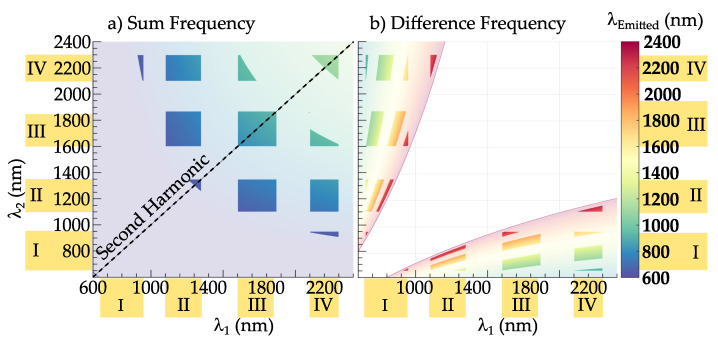
Simulation on the sum frequency generation (**a**) and difference frequency generation (**b**) in the wavelength range from 600 till 2400 nm, covering the biological windows NIR-I to IV, marked in yellow. The emitted wavelength can be read in the colorbar. The dashed line represents the second harmonic generation, a special case of the sum frequency generation when $\lambda_{1}=\lambda_{2}$. The areas with reduced opacity refer to all wavelength pairs of SFG/DFG that are lying outside of the biological windows.

We will now turn over to discuss the perspective of the NIR-to-NIR imaging approach using amplified fs-laser pulses to excite harmonic nanoparticles and taking our findings into account. In particular, we will consider the further frequency conversion concepts of sum frequency generation (SFG) and difference frequency generation (DFG). In these cases, two light pulses of different photon energies $\omega_{1},\omega_{2}$ are mixed in the nanoparticle and generate either the sum ($\omega_{1}+\omega_{2}$) or the difference frequency ($\omega_{1}-\omega_{2}$) at the output. The sum and difference frequency conversion in HNPs has already been demonstrated in previous studies [44,45] and appears concurrently to harmonic generation, as shown in [45], thus extending the concept of multi-harmonic imaging, which was previously limited to the simultaneous acquisition of SHG and THG [17,25]. SFG and DFG give even more freedom in the tailoring of the excitation and emission according to the biological windows. Figure 5 shows a simulation of these second order nonlinear processes combined with the information of the biological window NIR-I to IV. In particular, SFG is shown in Figure 5a, where the black dotted line represents the SHG, a special case of the sum frequency when $\lambda_{1}=\lambda_{2}$, while DFG is displayed in Figure 5b. The result gives an idea on the full potential of the technique, which experimentally is far from being fully exploited and implemented in a microscopy technique, and new combinations in excitation and emission can be expected. For example, actually fluorescent nanoprobes cannot emit in NIR-IV [4], but via DFG this would be now possible.

We here underline that these considerations are valid when employing biological tissue having extinction coefficient with similar minima as the one of the skin, i.e., the concept of biological windows can be applied (cf. Figure 1). At the same time, it is important to highlight that the concept is even more powerful for tissue that is not water-based. For examples, the extinction coefficient of bones [10] shows a monotonous decrease of the absorption coefficient from the visible to 1750 nm and remains nearly constant up to 2500 nm. Typical absorption peaks of water around 1450 and 1900 nm do not show-up, so that in this case harmonic nanoparticles can be used in a much larger range of wavelengths, and many more pairing configurations may be applied.

## 5. Conclusions

Harmonic nanoparticles pumped by a regeneratively amplified femtosecond laser system close the gap of NIR-to-NIR biomarkers that can be excited in the NIR-III and/or NIR-IV biological windows, but also can be used as emitters in these regions. Their emission feature turns out to be very efficient for deep tissue imaging and can be increased further by the simple use of larger pulse energies in the NIR. From the perspective of applications in bio- and life sciences, it is noteworthy to add that harmonic polar oxide nanoparticles are biocompatible and offer the possibility for surface functionalization [16]. This potentially enables site specific labeling, e.g., at tumor cells, but also targeted optical access to trigger photochemical processes with very high spatial and temporal resolution—with particular emphasis to the fairly unexplored NIR-III and NIR-IV regions. At the same time, the imaging technology is based on state-of-the-art microscopic and laser systems with an easy optical coupling path, which is the prerequisite for a wider range of applications. The investigation of emission in the NIR-III and NIR-IV requires detectors with sensitivity in the respective spectral regions and are the starting point for upcoming studies to further evaluate the impact of the concept and added-value for bio- and life sciences.

## Data Availability

The data presented in this study are available on request from the corresponding author.

## Abbreviations

The following abbreviations are used in this manuscript:

NIR	Near-Infrared
HNP	Harmonic Nanoparticles
SHG	Second Harmonic Generation
THG	Third Harmonic Generation
SNR	Signal to Noise Ratio
OPA	Optical Parametric amplifier
SFG	Sum Frequency Generation
DFG	Difference Frequency Generation
